# NAT10 Promotes Osteogenic Differentiation of Mesenchymal Stem Cells by Mediating N4-Acetylcytidine Modification of Gremlin 1

**DOI:** 10.1155/2021/8833527

**Published:** 2021-04-12

**Authors:** Zhenbiao Zhu, Xiaowei Xing, Shisi Huang, Yuanyuan Tu

**Affiliations:** ^1^Department of Orthopaedics, Central South University Xiangya School of Medicine Affiliated Haikou Hospital, Haikou, Hainan, China; ^2^Department of Oncology, Central South University Xiangya School of Medicine Affiliated Haikou Hospital, Haikou, Hainan, China

## Abstract

**Objective:**

To investigate the function of NAT10 in mesenchymal stem cell (MSC) osteogenic differentiation and study the mechanism by which NAT10 affects MSC osteogenesis by mediating Gremlin 1 N4-acetylcytidine (ac^4^C) modification.

**Methods:**

Osteogenic differentiation of MSCs was induced, and the osteogenic ability was evaluated with alizarin red S (ARS) and alkaline phosphatase (ALP) assays. The NAT10 expression level during MSC osteogenesis was measured by western blot (WB). MSCs were transfected with lentiviruses to inhibit (Sh-NAT10) or overexpress NAT10 (Over-NAT10), and the osteogenic differentiation ability was assessed by ARS, ALP, and osteogenic gene marker assays. *β*-Catenin, Akt, and Smad signaling pathway component activation levels were assessed, and the expression levels of key Smad signaling pathway molecules were determined by PCR and WB. The Gremlin 1 mRNA ac^4^C levels were analyzed using RIP-PCR, and the Gremlin 1 mRNA degradation rate was determined. Sh-Gremlin 1 was transfected to further investigate the role of NAT10 and Gremlin 1 in MSC osteogenesis.

**Results:**

During MSC osteogenesis, NAT10 expression, ARS staining, and the ALP level gradually increased. Decreasing NAT10 expression inhibited, and increasing NAT10 expression promoted MSC osteogenic differentiation. NAT10 affected the BMP/Smad rather than the Akt and *β*-Catenin signaling pathway activation by regulating Gremlin 1 expression. The Gremlin 1 mRNA ac^4^C level was positively regulated by NAT10, which accelerated Gremlin 1 degradation. Sh-Gremlin 1 abolished the promotive effect of NAT10 on MSC osteogenic differentiation.

**Conclusion:**

NAT10 positively regulated MSC osteogenic differentiation through accelerating the Gremlin 1 mRNA degradation by increasing its ac^4^C level. These results may provide new mechanistic insight into MSC osteogenesis and bone metabolism *in vivo*.

## 1. Introduction

Mesenchymal stem cells (MSCs), first characterized in 1976, are stem cells with multipotency *in vivo* [1]. In addition to their strong regulatory ability as components of the immune system, MSCs possess the capacity for trilineage differentiation, namely, osteogenic differentiation, chondrogenic differentiation, and adipogenic differentiation [2, 3]. As the major precursors of osteoblasts, MSCs contribute greatly to bone metabolism in the human body [4]. Moreover, MSCs are widely used in tissue engineering applications, especially bone repair [5]. Previously, many researchers have investigated the mechanism of MSC osteogenic differentiation, which, however, remains unclear.

The functions of MSCs are controlled by various kinds of RNA modifications [6]. Recently, N^4^-acetylcytidine (ac^4^C) modification was found to be widely distributed on RNA in human cells [7]. Messenger RNAs (mRNAs) modified by ac^4^C exhibit differences in their stability and degradation rate, therefore affecting the expression of genes and the subsequent functions of cells [8]. N-Acetyltransferase 10 (NAT10) is a critical RNA acetyltransferase mediating ac^4^C modification of mRNA [9]. However, whether NAT10 affects the osteogenic differentiation capacity of MSCs through ac^4^C modification is unknown.

Gremlin 1, a highly conserved 184 amino acid protein, is a member of the cystine knot superfamily. An antagonist of bone morphogenetic protein (BMP), Gremlin 1 shows a strong ability to inhibit osteogenic differentiation of MSCs by targeting BMP2 and BMP4, which play an important role in bone development [10]. However, the upstream regulatory mechanism of Gremlin 1 expression has never been studied.

In this study, we investigated the role of NAT10 in MSC osteogenesis. Our results showed that NAT10 increased the ac^4^C modification of Gremlin 1 mRNA and accelerated its degradation rate, which activated the BMP/Smad signaling pathway, positively regulating the osteogenic differentiation ability of MSCs. These results emphasize the importance of NAT10-mediated ac^4^C modification on the osteogenic differentiation of MSCs and may provide new insight into the clinical use of MSCs in bone tissue engineering.

## 2. Materials and Methods

### 2.1. Culture of Human Bone MSCs

Human bone marrow-derived MSCs were purchased from PromoCell (Heidelberg, Germany) and cultured in growth medium (low-glucose DMEM (Gibco) containing 10% fetal bovine serum (FBS; Gibco), 1% penicillin-streptomycin, and 1% glutamine) in 75 cm^2^ culture dishes at 37°C in 5% CO_2_. The culture medium was changed every 3 days. All experiments were performed using cells at passages 3-5.

### 2.2. Induction of Osteogenic Differentiation

For osteoblastic differentiation, MSCs were cultured in osteoblast differentiation medium (10% fetal bovine serum (Gibco; Thermo Fisher Scientific, Inc.), 10 nM dexamethasone, 10 mM *β*-glycerophosphate, 50 *μ*g/ml ascorbic acid, 1% L-glucose, and 1% penicillin-streptomycin) from day 0 to day 14. The osteogenic induction medium was replaced every 2 days.

### 2.3. ARS Staining

MSCs were subjected to osteogenic differentiation for 0-14 days. The medium was discarded, and the cells were washed with PBS 3 times, fixed with 4% paraformaldehyde for 30 minutes at 37°C in the dark and stained with 40 mM ARS (Sigma-Aldrich) for 15 min at room temperature. Calcification nodules were observed, and images were acquired using an inverted light microscope (magnification, 40x).

For ARS quantification, alizarin red was extracted with 10% cetylpyridinium chloride monohydrate (CPC, Meilunbio) for 60 min at room temperature with shaking. The absorbance of the extracted supernatant was measured at 562 nm.

### 2.4. ALP Staining and Activity Assay

ALP staining was performed according to the manufacturer's instructions from 0 to 14 days after initiation of osteogenic induction. In brief, cells were washed with PBS 3 times for 5 min each, and an appropriate amount of BCIP/NBT staining solution was added. Samples were incubated at room temperature in the dark for 20 min. Cells were washed twice with distilled water to terminate the reaction. Images of stained cells were acquired under a microscope.

ALP activity was measured by using a modified Great Escape SEAP Chemiluminescence Assay (BD Clontech, Mountain View, CA) based on the original secreted alkaline phosphatase (SEAP) enzymatic reporter assay, which does not require cell lysis. MSCs were lysed with lysis buffer and centrifuged for 5 min. Then, 5 *μ*l of cell lysate supernatant and 20 *μ*l of ALP substrate were mixed and incubated for 60 min in the dark. The results were obtained with a Hitachi 7060C Automatic Biochemical Analyzer.

### 2.5. Western Blot Analysis

Total protein was extracted from MSCs with lysis buffer (Beyotime, Haimen, China) containing phosphatase and protease inhibitors (Roche, Mannheim, Germany). A total of 30 *μ*g of protein was loaded per lane for electrophoresis. Extracted proteins were separated on a 10% SDS-PAGE gel. After the proteins were transferred to PVDF membranes (EMD Millipore), the membranes were blocked with 5% skim milk at room temperature for 2 h. The membranes were then incubated with primary antibodies at 4°C overnight and with secondary antibodies at room temperature for 2 h. Bands were visualized with an enhanced chemiluminescence (ECL) detection kit (Amersham; GE Healthcare) and analyzed with ImageJ Software (version 1.38; National Institutes of Health). The following antibodies were used: rabbit monoclonal anti-NAT10 (1 : 1000, ab194297, Abcam), rabbit monoclonal anti-GAPDH (1 : 1000, 2118, Cell Signaling Technology), rabbit monoclonal anti-phospho (p)-*β*-Catenin (Ser675) (1 : 1000, 4176, Abcam), rabbit polyclonal anti-*β*-Catenin (1 : 1000, ab16051, Abcam), rabbit polyclonal anti-p-Akt (Thr308) (1 : 1000, ab38449, Abcam), rabbit monoclonal anti-Akt (1 : 1000, 4685, Cell Signaling Technology), rabbit monoclonal anti-p-Smad1 (Ser463/465)/Smad5 (Ser463/465)/Smad9 (Ser465/467) (1 : 1000, 13820, Cell Signaling Technology), rabbit monoclonal anti-Smad1 (1 : 1000, ab126761, Abcam), rabbit polyclonal anti-Gremlin 1 (1 : 1000, ab22138, Abcam), rabbit monoclonal anti-ERK1/2 (1 : 1000, ab184699, Abcam), rabbit monoclonal anti-p-ERK1 (T202)/anti-p-ERK2 (T185) (1 : 1000, ab201015, Abcam), rabbit monoclonal anti-Smad2/3 (1 : 1000, ab202445, Abcam), rabbit polyclonal p-Smad2/3 (T8) (1 : 1000, ab272332, Abcam), rabbit monoclonal Jagged1 (Jag1) (1 : 1000, ab109536, Abcam), rabbit monoclonal Notch1 (1 : 1000, ab52627, Abcam), rabbit monoclonal anti-Gli1 (1 : 1000, 134906, Abcam), rabbit monoclonal anti-Sonic Hedgehog (Shh) (1 : 1000, ab53281, Abcam), and a goat secondary antibody (1 : 3000, 7074 or 7076, Cell Signaling Technology).

### 2.6. Lentiviral Infection

First, three siRNAs targeting NAT10 and Gremlin 1 were designed, and the most effective siRNA was selected for generation of lentiviruses. The sequence used for NAT10 knockdown was 5′-GGCCAAACAAGAACCCAAACA-3′, the sequence used for Gremlin 1 knockdown was 5′-GCATGTGACGGAGCGCAAATA-3′, and the sequence used as the knockdown negative control was 5′-TTCTCCGAACGTGTCACGTTTC-3′. For the generation of overexpression lentiviruses, the complete nucleotide sequence of NAT10 was synthesized. Both the knockdown and overexpression lentiviruses were generated by GenePharma Company. Lentiviruses were incubated with MSCs and 5 *μ*g/ml polybrene for 24 h at an MOI of 30.

### 2.7. Quantitative Real-Time PCR (qRT-PCR)

Total RNA was extracted from cells using TRIzol reagent (Invitrogen, Carlsbad, CA, USA). The RNA quantity and quality were assessed a NanoDrop spectrophotometer (Thermo Fisher Scientific, Inc., Austin, TX, USA). Then, 0.5 mg of RNA was reverse transcribed to cDNA using a High Capacity cDNA Reverse Transcription Kit (Applied Biosystems, NY, USA). Target genes were detected by qRT-PCR using SYBR Green Master reagents (F. Hoffmann-La Roche AG, Basel, Switzerland). Glyceraldehyde-3-phosphate dehydrogenase (GAPDH) was used as the internal control for measuring gene expression levels. The relative expression levels of target genes were evaluated using the 2^-*ΔΔ*CT^ method. The primer sequences are shown in Table 1.

### 2.8. ac^4^C RNA Immunoprecipitation-PCR (RIP-PCR)

The ac^4^C RIP assay was performed using an EZ-Magna RIP™ RNA-Binding Protein Immunoprecipitation Kit (Sigma-Aldrich, 17-701) according to the manufacturer's instructions. In brief, RNA was extracted and then chemically sheared into fragments of 200 or fewer nucleotides. The RNA fragments were incubated with anti-ac^4^C antibody- or IgG-conjugated Protein A/G magnetic beads at 4°C overnight. Then, the magnetic beads were collected, and the bound ac^4^C-modified RNA was eluted for analysis by qRT-PCR as described above. Equal amounts of RNA fragments not subjected to immunoprecipitation were used as the input control.

### 2.9. RNA Stability Assays

MSCs were seeded in 12-well plates and treated with actinomycin D at a concentration of 20 *μ*g/ml for 0, 0.5, 1, and 2 h. After treatment, RNA was immediately extracted, and PCR was performed. The turnover rate and half-life of the target mRNA were calculated to analyze the degradation rate.

### 2.10. Statistical Analysis

SPSS 21.0 (IBM Corp., Armonk, NY, USA) was utilized for statistical analysis. Measurement data with a normal distribution and homogeneous variance are expressed as the mean ± standard deviation of the values from three independent experiments. An unpaired *t*-test was adopted to analyze differences between two experimental groups, while one-way analysis of variance (ANOVA) followed by Tukey's post hoc test was utilized to compare data among multiple groups. Repeated measures ANOVA followed by the Bonferroni post hoc test was used to analyze data among multiple groups at different time points. A *P* value of <0.05 was considered to indicate a statistically significant difference.

## 3. Results

### 3.1. NAT10 Was Upregulated during Osteogenic Differentiation of MSCs

**Figure 1:**
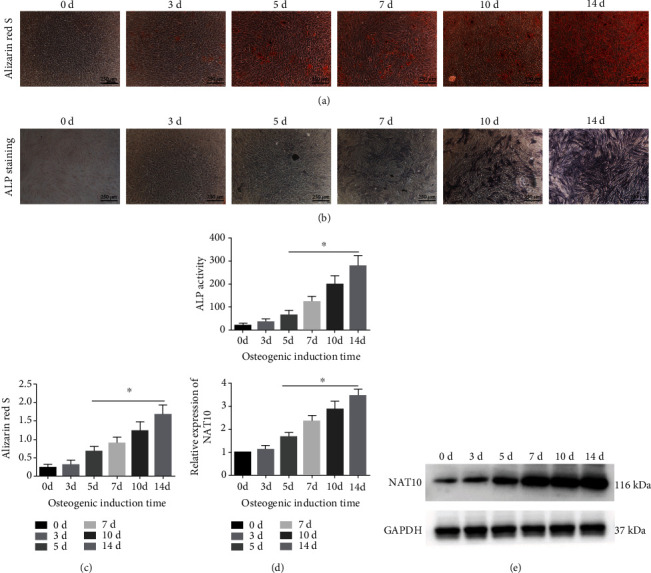
NAT10 was upregulated during osteogenic differentiation of MSCs. (a) Staining of ARS showed increased calcium nodule formation from day 0 to day 14 of induction. Scale bar = 250 *μ*m. (b) The ALP staining intensity increased from day 0 to day 14 of induction. Scale bar = 250 *μ*m. (c) The quantification of ARS staining increased from day 0 to day 14 of induction. (d) The ALP activity increased from day 0 to day 14 of induction. (e) The protein expression of NAT10 increased gradually from day 0 to day 14 of induction. ∗ indicates *P* < 0.05. All experiments were repeated three times.

First, we induced MSCs to undergo osteogenic differentiation and evaluated the osteogenic differentiation capacity at different time points by ARS and ALP assays. The number of calcium nodules that could be stained by ARS increased from day 0 to day 14 after induction (Figures 1(a) and 1(c)). The results of the ALP assay were consistent with those of ARS staining (Figures 1(b) and 1(d)). Accordingly, the protein level of NAT10 increased in MSCs undergoing osteogenic differentiation, and its expression pattern was similar to that demonstrated by the ARS and ALP assays (Figure 1(e)).

### 3.2. NAT10 Positively Regulated the Osteogenic Capacity of MSCs

**Figure 2:**
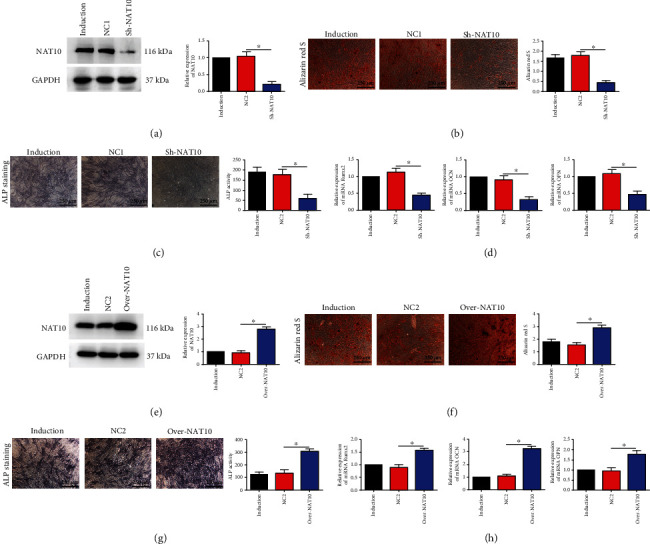
NAT10 positively regulated the osteogenic capacity of MSCs. (a) Sh-NAT10 significantly decreased the protein level of NAT10. (b) ARS staining and quantification were decreased in the Sh-NAT10 group compared to the negative control (NC) lentivirus group. Scale bar = 250 *μ*m. (c) ALP staining and activity were decreased in the Sh-NAT10 group compared to the NC lentivirus group. Scale bar = 250 *μ*m. (d) RUNX2, OPN, and OCN mRNA expression was decreased in the Sh-NAT10 group. (e) Over-NAT10 significantly increased the protein level of NAT10. (f) ARS staining and quantification were increased in the Over-NAT10 group compared to the NC lentivirus group. Scale bar = 250 *μ*m. (g) ALP staining and activity were increased in the Over-NAT10 group compared to the NC lentivirus group. Scale bar = 250 *μ*m. (h) RUNX2, OPN, and OCN mRNA expression was increased in the Over-NAT10 group. ∗ indicates *P* < 0.05. All experiments were repeated three times. The induction group indicates MSCs undergoing osteogenic differentiation without other treatment. NC1 indicates control lentivirus-transfected MSCs undergoing osteogenic differentiation.

To further explore the role of NAT10 in the osteogenic differentiation of MSCs, we constructed lentiviruses expressing an shRNA for NAT10 knockdown (Sh-NAT10). The following experiments to study the role of NAT10 in osteogenic differentiation were conducted after 14 days of induction. The results of western blot (WB) confirmed that this shRNA effectively inhibited the expression of NAT10 in MSCs (Figure 2(a)). After this inhibition, NAT10 expression, the ARS staining level and quantification, and ALP activity and staining intensity in MSCs were significantly reduced compared to those in both the induction group and the control lentivirus group (Figures 2(b) and 2(c)). *RUNX2*, *OCN*, and *OPN* are marker genes commonly used to monitor MSC osteogenesis. The mRNA expression levels of all these markers were decreased in the Sh-NAT10 group (Figure 2(d)). These results indicated that knockdown of NAT10 inhibited the osteogenesis of MSCs.

We further generated another lentivirus to overexpress NAT10 (Over-NAT10). The results of WB confirmed that infection with Over-NAT10 lentivirus produced effective overexpression of NAT10 in MSCs (Figure 2(e)). In addition, ARS and ALP staining was much stronger in the Over-NAT10 group than in the induction and control lentivirus groups (Figures 2(f) and 2(g)). Accordingly, the mRNA levels of *RUNX2*, *OCN*, and *OPN* were significantly increased (Figure 2(h)). In summary, we determined that NAT10 positively regulated the osteogenic capacity of MSCs.

### 3.3. NAT10 Regulated the Activation of the Smad1/5/9 Signaling Pathway and the Expression of Gremlin 1 through ac^4^C Modification

**Figure 3:**
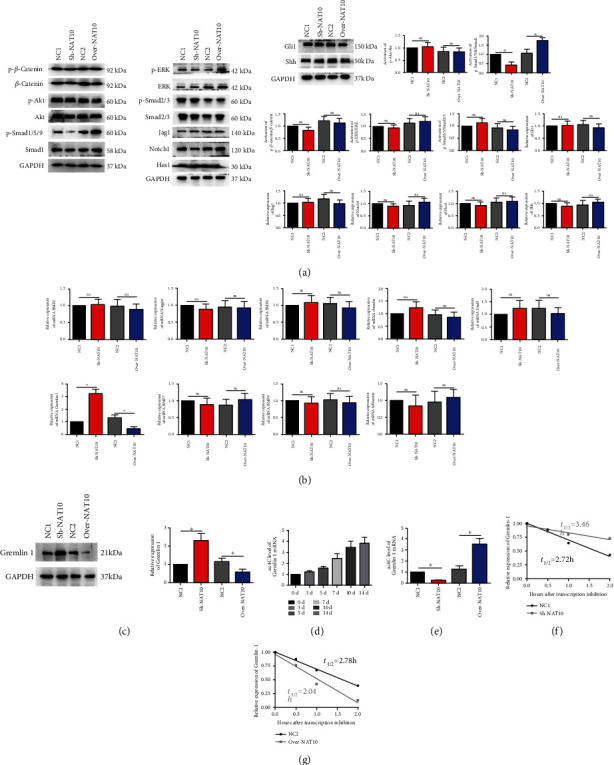
NAT10 regulated the activation of the Smad1/5/9 signaling pathway and the expression of Gremlin 1 through ac^4^C modification. (a) The phosphorylation levels of Smad1/5/9 signaling pathway components were decreased in the Sh-NAT10 group but increased in the Over-NAT10 group. The activation levels of *β*-Catenin, Akt, Smad2/3, FGF/ERK, Noggin, and Hedgehog signaling pathway components remained stable in the different groups. (b) The mRNA level of Gremlin 1 was increased in the Sh-NAT10 group but decreased in the Over-NAT10 group. The mRNA levels of BMP2, Noggin, BMP4, BMP7, BMP9, Dand5, Follistatin, and Chordin remained stable in the different groups. (c) The protein level of Gremlin 1 was increased in the Sh-NAT10 group but decreased in the Over-NAT10 group. (d) The ac^4^C level in Gremlin 1 mRNA increased gradually from day 0 to day 14 of MSC osteogenesis. (e) The ac^4^C level in Gremlin 1 was lower in the Sh-NAT10 group and higher in the Over-NAT10 group than in the NC group. (f) The degradation rate of Gremlin 1 mRNA was slower in the Sh-NAT10 group than in the NC group. (g) The degradation rate of Gremlin 1 mRNA was higher in the Over-NAT10 group than in the NC group. ∗ indicates *P* < 0.05. All experiments were repeated three times.

We then determined the activation levels of the *β*-Catenin, Akt and Smad1/5/9, Smad2/3, FGF/ERK, Noggin, and Hedgehog signaling pathways, which have been reported to be important in the osteogenesis of MSCs. Although the activation levels of the *β*-Catenin, Akt, Smad2/3, FGF/ERK, Noggin, and Hedgehog pathways remained unchanged in MSCs after knockdown or overexpression of NAT10, activation of the Smad1/5/9 signaling pathway was significantly decreased in the Sh-NAT10 group but increased in the Over-NAT10 group (Figure 3(a)), indicating that NAT10 positively regulated the activation of the Smad1/5/9 signaling pathway. The Smad1/5/9 signaling pathway is activated by BMPs. Thus, we further explored the mRNA levels of multiple BMP family members and their antagonists. The results demonstrated that the mRNA levels of most BMPs and BMP antagonists remained stable after modulation of NAT10 expression (Figure 3(b)). However, the mRNA level of Gremlin 1, an antagonist of BMP2 and 4, was significantly increased in the Sh-NAT10 group but decreased in the Over-NAT10 group (Figure 3(b)). Moreover, the results of WB demonstrated that the protein level of Gremlin 1 was consistent with its mRNA level (Figure 3(c)).

Previous research demonstrated that NAT10 mediates ac^4^C modification and then affects gene expression. Therefore, we measured the ac^4^C level on Gremlin 1 mRNA. The results of ac^4^C RIP-PCR showed that the level of ac^4^C on Gremlin 1 mRNA increased during MSC osteogenic differentiation from day 0 to day 14 (Figure 3(d)). In addition, inhibition of NAT10 expression decreased the ac^4^C level on Gremlin 1 mRNA in MSCs, and overexpression of NAT10 significantly increased the ac^4^C level on Gremlin 1 mRNA (Figure 3(e)). Moreover, inhibition of NAT10 expression markedly reduced the degradation rate of Gremlin 1 mRNA (Figure 3(f)). In contrast, overexpression of NAT10 in MSCs accelerated the degradation of Gremlin 1 mRNA (Figure 3(g)).

### 3.4. NAT10 Inhibited the Expression of Gremlin 1 to Modulate the Activation of the Smad1/5/9 Signaling Pathway and the Osteogenic Capacity of MSCs

**Figure 4:**
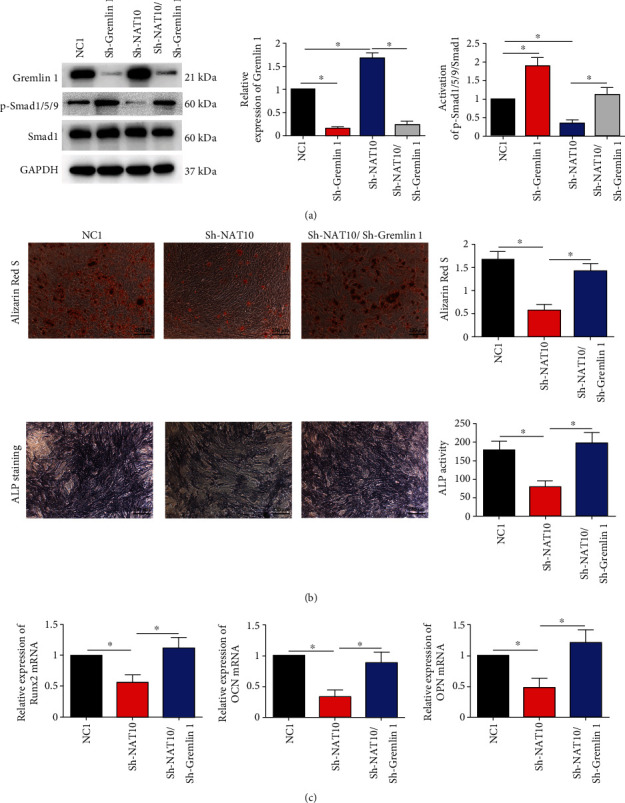
NAT10 inhibited the expression of Gremlin 1 to modulate the activation of the Smad1/5/9 signaling pathway and osteogenic capacity of MSCs. (a) Sh-Gremlin 1 combined with Sh-NAT10 restored the phosphorylation level of Smad1/5/9 compared with that in the Sh-NAT10 group. (b) ARS and ALP assays demonstrated that Sh-Gremlin 1/Sh-NAT10 rescued the osteogenic capacity of MSCs compared with that of the Sh-NAT10 group. Scale bar = 250 *μ*m. (c) Sh-Gremlin 1 combined with Sh-NAT10 restored the mRNA expression levels of RUNX2, OPN, and OCN compared with those in the Sh-NAT10 group. ∗ indicates P < 0.05. All experiments were repeated three times.

We further constructed lentiviruses expressing an shRNA for gremlin 1 (Sh-Gremlin 1) knockdown. The results of WB demonstrated that treatment with Sh-Gremlin 1 combined with Sh-NAT10 restored the activation level of Smad1/5/9 compared with that in Sh-NAT10 MSCs (Figure 4(a)). Accordingly, the results of ARS staining and ALP assays and the mRNA levels of osteogenesis markers demonstrated that treatment with Sh-Gremlin 1 combined with Sh-NAT10 rescued the osteogenic capacity of MSCs compared with that of Sh-NAT10 MSCs (Figure 4(b) and 4(c)). Therefore, we concluded that NAT10 regulated the activation of the Smad1/5/9 signaling pathway by modulating the expression of Gremlin 1.

## 4. Discussion

MSCs, also called multipotent stromal cells, contribute greatly to bone formation and development [11]. As the precursors of osteoblasts, MSCs undergo osteogenic differentiation in specific microenvironments and regulate the homeostasis of bone metabolism *in vivo* [12]. Moreover, due to their lower immunogenicity and stronger osteogenic ability than other stem cells, MSCs are used as seed cells in tissue engineering and regenerative medicine applications, such as bone defect repair [13, 14]. In addition, previous studies have demonstrated that decreasing the osteogenic differentiation ability of MSCs leads to osteoporosis and low bone mass in other kinds of diseases [15–17]. Therefore, illuminating the detailed mechanisms of MSC osteogenic differentiation could not only promote the comprehensive clinical use of MSCs but also facilitate investigating the pathogenesis of bone-related diseases.

RNA modification, including N^6^-methyladenosine, N^1^-methyladenosine, and 5-methylcytosine modifications, is a critical regulatory mechanism for protein expression and cell function [18]. Specifically, N^6^-methyladenosine plays an important role in mediating the functions of MSCs, including osteogenic differentiation [19]. Recently, ac^4^C was proven to be conserved and extensively distributed on mRNAs, contributing to their transcription and translation [7]. This modification ultimately affects various functions in various cells. NAT10, an RNA acetyltransferase, is responsible for ac^4^C modification of RNA [20]. However, whether NAT10-mediated ac^4^C modification of RNA affects the osteogenic differentiation of MSCs is unclear. To study this issue in depth, we evaluated NAT10 expression during the osteogenic differentiation of MSCs and found that the NAT10 expression profile was positively correlated with the ARS staining and ALP levels, suggesting the functional role of NAT10 in MSC osteogenesis. In addition, inhibition of NAT10 expression suppressed the osteogenic differentiation ability of MSCs, and constitutive overexpression of NAT10 in MSCs significantly promoted their osteogenic differentiation ability. To our knowledge, these results are the first to demonstrate that NAT10 positively regulates the osteogenesis of MSCs.

The second focus of our study was the mechanism by which NAT10 affects MSC osteogenic differentiation. Previous studies have proven that MSC osteogenic differentiation is controlled by various kinds of signaling pathways, such as the WNT/*β*-catenin, BMP/Smad, and Akt pathways [21]. Therefore, we first determined which signaling pathway was modulated by NAT10. Western blot analysis indicated that only the BMP/Smad signaling pathway was positively modulated by NAT10 in MSCs during osteogenic differentiation. In contrast, the activity levels of the WNT/*β*-Catenin and Akt signaling pathways remained unchanged, indicating that NAT10 regulated the osteogenic differentiation of MSCs mainly through the BMP/Smad signaling pathway. BMPs, including BMP2, BMP4, BMP7, and BMP9, bind to BMPR1 or BMPR2 and then phosphorylate and activate Smad1/5/9 downstream, which in turn promotes MSC osteogenesis [22]. BMP antagonists, including Noggin and Gremlin 1, inhibit the functions of BMPs and then suppress MSC osteogenesis [23]. In our study, we found that the expression levels of BMP2, BMP4, BMP7, BMP9, and Noggin were not affected by NAT10 and that Gremlin 1 mRNA and protein expression was negatively regulated by NAT10. Gremlin 1, a member of the cystine knot superfamily, is a natural antagonist of BMPs [24]. A recent study demonstrated that inhibition of Gremlin 1 expression promoted osteogenic differentiation of MSCs [25]. Moreover, we found that the promotive effect of NAT10 on MSC osteogenesis was abolished by Sh-Gremlin 1. Therefore, we concluded that Gremlin 1 was the downstream target of NAT10 and that NAT10 promoted MSC osteogenesis by inhibiting the expression of Gremlin 1.

A further question was whether NAT10 regulates the expression of Gremlin 1 through ac^4^C modification. A previous study indicated that ac^4^C RNA modification affected the stability of the target mRNA [8]. Herein, we found that ac^4^C modification of Gremlin 1 mRNA was gradually increased from day 0 to day 14 of osteogenic differentiation and that the ac^4^C level was positively correlated with NAT10 expression and MSC osteogenesis. In addition, the ac^4^C level on Gremlin 1 mRNA decreased after inhibition of NAT10 expression in MSCs and increased after infection with Over-NAT10 lentivirus. These results indicated that NAT10 mediated the ac^4^C modification of Gremlin 1 mRNA. Moreover, the degradation of Gremlin 1 mRNA was slower in the Sh-NAT10 group but faster in the Over-NAT10 group than in the control group. Therefore, these results suggested that NAT10 promoted the ac^4^C modification of Gremlin 1 mRNA, which then accelerated Gremlin 1 mRNA degradation and finally inhibited its expression. However, contrary to our results, a study in 293T cells showed that NAT10 mediated ac^4^C modification of HIV transcripts by increasing mRNA stability [26]. We therefore suggest that NAT10-mediated ac^4^C modification may exert different effects on different cells or on different genes in the same cell, a possibility that needs further study. Previous studies have demonstrated that RNA modification could promote the mRNA degradation. For example, m^6^A modification on MYB and MYC mRNA 3′UTR could accelerate their degradation and promote leukemogenesis development [27]. However, how ac^4^C modification regulates mRNA degradation is still unknown. We suggest that ac^4^C modification mediated by NAT10 may also affect Gremlin 1 mRNA degradation through targeting to its 3′UTR sites, which needs further study in the future.

## 5. Conclusion

In this study, we demonstrated that NAT10 promoted the osteogenic differentiation of MSCs. This effect was mediated by an increase in the ac^4^C modification level on Gremlin 1 mRNA, which accelerated its degradation, thus reducing the level of Gremlin 1 during osteogenic differentiation. These results may provide new insight into the mechanism of MSC osteogenesis that may contribute to the diagnosis and treatment of bone-related diseases such as osteoporosis.

## Figures and Tables

**Table 1:** Primer sequences.

Gene	Forward primer (5′-3′)	Reverse primer (5′-3′)
GAPDH	ATCCCATCACCATCTTCC	GAGTCCTTCCACGATACCA
RUNX2	ACTTCCTGTGCTCGGTGCT	GACGGTTATGGTCAAGGTGAA
Osteocalcin (OCN)	TGAGAGCCCTCACACTCCTC	CGCCTGGGTCTCTTCACTAC
Osteopontin (OPN)	CTCCATTGACTCGAACGACTC	CAGGTCTGCGAAACTTCTTAGAT
BMP2	ACCCGCTGTCTTCTAGCGT	TTTCAGGCCGAACATGCTGAG
Noggin	CCATGCCGAGCGAGATCAAA	TCGGAAATGATGGGGTACTGG
BMP4	AAAGTCGCCGAGATTCAGGG	GACGGCACTCTTGCTAGGC
BMP7	TCGGCACCCATGTTCATGC	GAGGAAATGGCTATCTTGCAGG
BMP9	AGAACGTGAAGGTGGATTTCC	CGCACAATGTTGGACGCTG
Gremlin 1	CGGAGCGCAAATACCTGAAG	GGTTGATGATGGTGCGACTGT
Chordin	TTCGGCGGGAAGGTCTATG	ACTCTGGTTTGATGTTCTTGCAG
Dand5	AAGTGATCCAGGGGATGTGTA	GATGATTTCGGAGGCGTATGG
Follistatin	ACGTGTGAGAACGTGGACTG	CACATTCATTGCGGTAGGTTTTC

## Data Availability

Data supporting the findings of this manuscript are available from the corresponding author upon reasonable request.

## Supplementary Materials

Supplementary Materials Supplemental Figure 1: original strip of WB in this manuscript.

## References

[1] Pittenger M. F., Discher D. E., Péault B. M., Phinney D. G., Hare J. M., Caplan A. I. (2019). Mesenchymal stem cell perspective: cell biology to clinical progress. *npj Regenerative Medicine*.

[2] Wang M., Yuan Q., Xie L. (2018). Mesenchymal stem cell-based immunomodulation: properties and clinical application. *Stem Cells International*.

[3] Galipeau J., Sensebe L. (2018). Mesenchymal stromal cells: clinical challenges and therapeutic opportunities. *Cell Stem Cell*.

[4] Ambrosi T. H., Longaker M. T., Chan C. K. F. (2019). A revised perspective of skeletal stem cell biology. *Frontiers in Cell and Development Biology*.

[5] Wang J., Chen Z., Sun M. (2020). Characterization and therapeutic applications of mesenchymal stem cells for regenerative medicine. *Tissue & Cell*.

[6] Sui B. D., Zheng C. X., Li M., Jin Y., Hu C. H. (2020). Epigenetic regulation of mesenchymal stem cell homeostasis. *Trends in Cell Biology*.

[7] Jin G., Xu M., Zou M., Duan S. (2020). The processing, gene regulation, biological functions, and clinical relevance of N4-acetylcytidine on RNA: a systematic review. *Mol Ther Nucleic Acids*.

[8] Arango D., Sturgill D., Alhusaini N. (2018). Acetylation of cytidine in mRNA promotes translation efficiency. *Cell*.

[9] Sleiman S., Dragon F. (2019). Recent advances on the structure and function of RNA acetyltransferase Kre 33/NAT10. *Cell*.

[10] Gooding S., Leedham S. J. (2020). Gremlin 1 — small protein, big impact: the multiorgan consequences of disruptedBMPantagonism†. *The Journal of Pathology*.

[11] Chu D. T., Phuong T., Tien N. (2020). An update on the progress of isolation, culture, storage, and clinical application of human bone marrow mesenchymal stem/stromal cells. *International Journal of Molecular Sciences*.

[12] Xu G. P., Zhang X. F., Sun L., Chen E. M. (2020). Current and future uses of skeletal stem cells for bone regeneration. *World J Stem Cells*.

[13] Favreau H., Pijnenburg L., Seitlinger J. (2020). Osteochondral repair combining therapeutics implant with Mesenchymal stem cells spheroids. *Nanomedicine*.

[14] Lee Y. C., Chan Y. H., Hsieh S. C., Lew W. Z., Feng S. W. (2019). Comparing the osteogenic potentials and bone regeneration capacities of bone marrow and dental pulp mesenchymal stem cells in a rabbit calvarial bone defect model. *International Journal of Molecular Sciences*.

[15] Wang Q., Li Y., Zhang Y. (2017). LncRNA MEG3 inhibited osteogenic differentiation of bone marrow mesenchymal stem cells from postmenopausal osteoporosis by targeting miR-133a-3p. *Biomedicine & Pharmacotherapy*.

[16] Tang Y., Xie H., Chen J. (2013). ActivatedNF-*κ*Bin bone marrow mesenchymal stem cells from systemic lupus erythematosus patients inhibits osteogenic differentiation through downregulating Smad signaling. *Stem Cells and Development*.

[17] Zhuang W., Ge X., Yang S. (2015). Upregulation of lncRNA MEG3 promotes osteogenic differentiation of mesenchymal stem cells from multiple myeloma patients by targeting BMP4 transcription. *Stem Cells*.

[18] Roundtree I. A., Evans M. E., Pan T., He C. (2017). Dynamic RNA modifications in gene expression regulation. *Cell*.

[19] Wu Y., Xie L., Wang M. (2018). Mettl3-mediated m^6^A RNA methylation regulates the fate of bone marrow mesenchymal stem cells and osteoporosis. *Nature Communications*.

[20] Ito S., Horikawa S., Suzuki T. (2014). Human NAT10 is an ATP-dependent RNA acetyltransferase responsible for *N*^4^-acetylcytidine formation in 18 S ribosomal RNA (rRNA). *The Journal of Biological Chemistry*.

[21] Chen Q., Shou P., Zheng C. (2016). Fate decision of mesenchymal stem cells: adipocytes or osteoblasts?. *Cell Death and Differentiation*.

[22] Carreira A. C., Zambuzzi W. F., Rossi M. C., Filho R. A., Sogayar M. C., Granjeiro J. M. (2015). Bone morphogenetic proteins: promising molecules for bone healing, bioengineering, and regenerative medicine. *Vitamins and Hormones*.

[23] Brazil D. P., Church R. H., Surae S., Godson C., Martin F. (2015). BMP signalling: agony and antagony in the family. *Trends in Cell Biology*.

[24] Li D., Yuan D., Shen H., Mao X., Yuan S., Liu Q. (2019). Gremlin-1: an endogenous BMP antagonist induces epithelial-mesenchymal transition and interferes with redifferentiation in fetal RPE cells with repeated wounds. *Molecular Vision*.

[25] Hu K., Sun H., Gui B., Sui C. (2017). Gremlin-1 suppression increases BMP-2-induced osteogenesis of human mesenchymal stem cells. *Molecular Medicine Reports*.

[26] Tsai K., Jaguva Vasudevan A. A., Martinez Campos C., Emery A., Swanstrom R., Cullen B. R. (2020). Acetylation of cytidine residues boosts HIV-1 gene expression by increasing viral RNA stability. *Cell Host & Microbe*.

[27] Weng H., Huang H., Wu H. (2018). METTL14 inhibits hematopoietic stem/progenitor differentiation and promotes leukemogenesis via mRNA m6A modification. *Cell Stem Cell*.

